# Programmatic Assessment: Tenets to Remember, Tensions to Resolve, Traps to Avoid, and Tips to Take on Board

**DOI:** 10.1007/s40670-026-02828-y

**Published:** 2026-07-23

**Authors:** Alison M Sturrock, Rakesh Patel

**Affiliations:** 1 University College London School of Life and Medical Sciences, UCL medical school, London, UK; 2 School of Medicine, St Mary’s University, London, UK

**Keywords:** Programmatic Assessment, Assessment for Learning, Assessment Validity and Reliability, Feedback and Learner Development, Trust in Assessment

## Abstract

This paper evaluates programmatic assessment (PA) as an educationally coherent framework for medical education, addressing longstanding paradoxes in traditional high stakes models where siloed performance data may fail to capture true learner competence. Programmatic assessment is a comprehensive, programme level strategy that uses multiple low stakes data points over time to inform high stakes progression decisions. While traditional summative assessments provide standardised, objective thresholds essential for national licensing, a growing body of longitudinal evidence indicates that the little and often approach of PA promotes deeper learning and improved retention of clinical skills.

In an era of disruption from Generative Artificial Intelligence (GenAI) and Large Language Models (LLMs), this paper explores how PA can be operationalised to enhance the validity of assessment decisions. We outline the core tenets of the model and resolve practical tensions, such as the balance between psychometrics and human judgement. We also introduce the concept of performance resilience, explaining how continuous feedback prepares students for high stakes clinical crises and national exams. Finally, we identify common implementation traps and offer ten practical tips, grounded in change management theory, to guide realistic institutional adoption. We conclude that programmatic assessment enhances learning, supports equity, and strengthens public trust in medical education.

## Introduction

“We would not consider telling a patient that their glucose level is far too high but fortunately their potassium level is far too low so on average they are okay [1].” Yet in medical education we frequently accept a similar contradiction: a student may fail to perform a neurological examination adequately but, because they excel in taking a haematology history, they are deemed competent overall. This paradox provides the context for this paper.

Such contradictions have persisted over time, shaping how students engage with assessment and influencing staff in ways that are not always educationally healthy. More importantly, they raise fundamental questions about the trustworthiness of assessment decisions, particularly given the level of trust society places in the medical profession to ensure that graduating doctors are genuinely competent [2, 3]. 

Many undergraduate medical programmes are described as ‘integrated’ with clinical and basic sciences taught in separate, siloed blocks. In such programmes, cross-compensation, the practice of allowing a high grade in one module to offset a failure in another, is often prohibited. Nevertheless, students may still make significant errors in high stakes end of year assessments, both written and clinical, indicating incomplete learning, yet progress without these deficiencies being systematically addressed. This observation does not constitute a critique of written or clinical assessments, nor an argument against high stakes assessment per se. Instead, it draws attention to assessment paradoxes in undergraduate medical education that require closer attention.

Programmatic assessment is defined as a comprehensive approach to evaluating learner competence through multiple low stakes assessments that, when considered collectively, inform high stakes decisions. It is not a single assessment method but a programme level strategy in which teaching, learning, and assessment are deliberately aligned.

Traditional summative assessments have considerable strengths, particularly in providing standardised, objective, and reliable measures of performance at a specific moment in time. They remain essential for national licensing and offer a clear binary threshold for entry into the profession. However traditional summative assessments often rely on modular structures, where students complete a discrete unit of study followed by a high stakes exam. This means the assessment is often siloed within the module, so performance data is rarely carried forward to inform future learning in different contexts. Programmatic assessment does not seek to replace these functions, but to situate them within a more coherent and educationally defensible approach.

These paradoxes have significant implications for trust. Learners must trust that assessment outcomes are fair, meaningful, and educationally sound, while society must trust that these outcomes reliably identify safe and competent practitioners [3]. 

The purpose of this paper is to revisit whether programmatic assessment is a more effective approach to evaluating learner performance than traditional assessment models [4, 5]. By drawing on evidence across an entire programme rather than a single assessment event, programmatic assessment addresses longstanding concerns about validity and reliability [6]. We also explore assumptions underpinning these psychometric concepts and examine the distinction between subjective and objective approaches to assessment [7–9]. 

While high stakes assessments provide a snapshot of performance, an accumulating body of evidence suggests that programmatic assessment offers a more robust framework for developing and judging long term competence [6, 10]. For instance, longitudinal studies have demonstrated that the ‘little and often’ approach to assessment leads to significantly better retention of clinical skills compared to traditional block-based testing [11]. By shifting emphasis from passing individual tests to continuous improvement, programmatic assessment supports deeper learning, improved retention of clinical knowledge, and earlier identification of learners requiring support [10, 11]. Moreover, by offering a transparent and comprehensive account of a student’s developmental journey, it has the potential to strengthen public trust in the profession’s certification processes [12]. 

The impetus for writing at this time arises from the practical challenges medical schools face when attempting to operationalise what remains, in many respects, an ideological and philosophical approach to assessment [6]. While the educational rationale for programmatic assessment is persuasive, the organisational and political case is less straightforward, largely because the empirical evidence base is still evolving and the economic implications are substantial. This reflection is also timely in light of emerging disruptors such as generative artificial intelligence (GenAI) and large language models (LLMs), which compel educators to reconsider what assessment is, what it measures, and how it should be designed and delivered [13, 14]. In this changing landscape, maintaining both public and learner trust in assessment decisions becomes ever more critical [3]. 

### Programmatic Assessment: Distinctions from a Programme of Assessment and from Progress Testing

Programmatic assessment is not the same as a programme of assessment. Programmatic assessment assumes that teaching, learning, and assessment are designed together at programme level; decisions about curriculum development take all these elements into account; and assessment becomes a programme wide activity with decoupled decision moments and rich feedback [4, 5]. It is led from the top, involves reviewing current practice, and depends on faculty effort to enact change [5, 6].

In contrast, a programme of assessment is usually developed from the bottom up. It often evolves within individual units or modules, led by staff responsible for their own portion of the curriculum. This work typically occurs at teacher level and may lack sufficient oversight from above. There is nothing inherently wrong with this approach, and it reflects how many university courses are organised. However, programmatic assessment requires greater engagement at programme level. The role of the Director becomes more than administrative or operational. It becomes strategic and demands educational leadership.

Programmatic assessment looks beyond measurement and psychometrics. These areas have long dominated discussions about assessment. Instead, programmatic assessment views assessment through the lens of learning. It seeks to create a holistic picture of a learner. Decisions about testing are therefore grounded in educational philosophy, not solely in statistics. From this perspective, feedback becomes the central outcome of an assessment event [4, 15, 16]. Failure is no longer a simple binary end point that may have little relevance for future performance. The idea of “feedback forwards” is therefore more prominent [15]. In contrast, coefficients such as Cronbach’s alpha may offer limited meaning for many students and staff [17]. This does not imply that psychometrics lack value. Rather, it highlights that assessment design has often focused on method over the development of adaptable, flexible, lifelong learners.

Programmatic assessment is also different from progress testing. Progress testing uses a repeated, comprehensive, graduate level exam delivered at regular intervals. It tracks the growth and retention of functional medical knowledge over time. It shifts attention from subject specific tests to a broad, integrated understanding. It also reinforces that meaningful learning develops gradually and that cramming is ineffective [18, 19]. While progress testing offers clear benefits, programmatic assessment provides additional advantages. These extend beyond test format to how assessment data are used to support learning [20, 21]. 

Programmatic assessment is not a new idea, and the literature supporting it continues to grow [4, 22]. The challenge lies in aligning its philosophy with the realities of high stakes summative assessment. Universities and regulators require robust procedures and clear standards [3, 23]. Assessment delivery has also tended to focus on isolated events rather than continuous data collection that supports ongoing feedback. Furthermore, curriculum change is difficult to implement. Changing assessment can be even more challenging, especially when staff fear that altering established systems may harm learners.

Despite these challenges, the educational rationale for shifting towards programmatic principles is increasingly recognised in medical education. Medical education increasingly recognises the need to focus on learning and outcomes, not only on teaching and process. There is an opportunity to design out redundancy and design in coherence across curriculum and assessment [24, 25]. There are also growing calls to involve learners in assessment design. Yet student perspectives are often absent from most stages of development and delivery, even though assessment outcomes have major consequences for them. To move the conversation forward and to plant the seeds of change, we outline the core principles of programmatic assessment below, highlight tensions to resolve, identify traps to avoid, and offer practical tips for navigating barriers and resistance [16]. 

### Tenets to Remember

To understand the tenets of programmatic assessment, several principles and an overarching philosophy of learning and feedback are central.

Programmatic assessment is a comprehensive approach to evaluating learner competence through continuous, low stakes assessments that, when considered collectively, inform high stakes decisions [3, 20]. Rather than relying on a single-point-in-time high stakes examinations, it draws on multiple data sources gathered over an extended period to provide a detailed account of student progress [6]. This shift towards a holistic and developmental model is the hallmark of programmatic assessment. It places greater emphasis on the ongoing evaluation of clinical, cognitive, and professional competencies over time.

Whereas traditional approaches rely predominantly on assessments of performance to make a final evaluation about attainment of learning (i.e. achieved or not achieved), programmatic assessment relies on multiple evidence sources across contexts. Many experienced educators already provide excellent formative feedback and monitor student progress informally. Programmatic assessment seeks to formalise and scale these individual best practices into a programme wide system that ensures no student’s developmental needs are overlooked. The aim is to evaluate areas for improvement and to feed forward [15, 16], supported by structured activities and faculty scaffolds.

Evidence from multiple assessments, across multiple days and learning contexts, is integrated to construct a rich, multifaceted picture of learner competence. Educators synthesise qualitative and quantitative data collected over time, enabling deeper insights into student progress and supporting sustained feedback and reflection.

A defining feature of programmatic assessment is the separation of assessment activities from immediate decisions about progression. Learners complete numerous assessments across a programme, generating a continuous flow of feedback that informs adjustments to teaching and learning strategies [4, 21]. The model incorporates tasks with varying levels of perceived consequence, encouraging consistent engagement and shifting the emphasis from isolated performances to cumulative learning. By engaging in frequent low stakes assessments across various contexts, learners build performance resilience. This cumulative evidence base ensures that by the time they reach a high stakes national exam their competence is well practised and stable, reducing the risk of failure due to pressure of the moment.

By emphasising feedback, reflection, and iterative improvement, students develop a clearer understanding of their strengths and areas for further development. This promotes active engagement with learning and encourages the growth of self-directed, lifelong learners [6, 16]. Ultimately, programmatic assessment moves beyond summative judgement, positioning assessment as a dynamic and integral component of the educational process. Transparent processes, accessible data, and clear criteria also help build trust among learners and the public [3]. 

### Tensions

Presenting these tenets on paper is one thing. Resolving the tensions between current practice and the practical implications of a purist implementation is another.

#### Psychometrics Versus Human Judgement

Many educators look to numbers for reassurance when making progression decisions. Others prefer human judgement and narrative evidence. Numerical outputs can provide comfort regardless of their true meaning and may escape scrutiny when validity is uncertain. There is also a tendency to believe that statistical outputs guarantee objectivity. This view overlooks the fact that tests such as OSCEs still rely on examiner judgement [26–28]. In this respect, an OSCE may be no more objective than a multisource feedback report judged on the same criteria. These misconceptions point to a broader issue: assessment literacy among faculty [29]. Developing this literacy is essential. It enables meaningful debate about assessment and brings underlying principles to the fore. 

#### Summative Dominance Versus Formative Richness

Summative assessments continue to dominate progression decisions in medical education, while formative assessments in workplace settings remain under used [4, 30] This imbalance is partly driven by misunderstandings about formative assessment, including misconceptions regarding the need for standardisation, the purpose of formative judgement and the underlying educational philosophy [31–33]. Traditional assessment practices have further diminished the perceived value of formative assessment, as learners are often provided with tasks that imitate summative examinations yet include irrelevant content or fail to replicate key examination conditions such as time limits, permitted resources or response formats [34, 35]. As a result, learner trust and engagement are reduced [36].

Despite this, formative assessment has a strong empirical and theoretical evidence base and remains central to effective programmatic assessment [15, 37, 38]. While national licensing examinations are necessarily summative in nature, programmatic assessment frameworks must create space for these requirements while continuing to prioritise formative learning [39, 40]. Addressing this tension requires deliberate and coherent integration between formative and summative assessment approaches [41, 42].

#### Work Ethic Versus Early Support

Some educators believe that students who fail at their first attempt have not worked hard enough. Others argue that these students did not receive adequate support early on [43, 44]. These positions are often shaped by educators’ own learning experiences and beliefs about self directed learning and learner responsibility [36, 45]. In contrast, many educators recognise a moral and professional obligation for medical schools to provide timely, structured support for learners in difficulty [46]. System level factors, including curriculum design and assessment practices, can also contribute to poor performance and repeated failure [10].

Programmatic assessment does not fully resolve this tension [4]. However, it explicitly prioritises early identification of concerns and targeted intervention, shifting emphasis from remediation after failure to support before it occurs [4]. Without addressing these underlying assumptions and improving curriculum and assessment coherence, cycles of remediation and repeated failure are unlikely to change [10, 46].

### Traps to Avoid

The tenets above help distinguish programmatic assessment from a programme of assessment. They also support early identification of learners in difficulty and promote rich narrative feedback. However, it is unrealistic to implement every tenet at once. Educators should avoid making “perfect” the enemy of “good enough” [3, 4]. 

#### Regulatory Reality

National licensing examinations are, by design, summative and often cannot be removed without legislative change. Some argue that having a high stakes assessment at the end of a programme prevents a system from being truly programmatic, but this is a misconception. Regulatory and legal requirements will always take precedence, and the existence of a single high stakes examination does not undermine a holistic, feedback rich curriculum grounded in programmatic principles. 

#### Inflexibility During Organisational Change

Transforming assessment practice inevitably involves negotiation and compromise. Senior leaders ultimately control the curriculum, which means change can be slow and constrained by competing priorities. A fully programmatic model may therefore not be achievable immediately, and insisting on a “pure” version is rarely productive. Progress depends not only on sound educational rationale but also on faculty development, organisational readiness, and economic feasibility. Humans naturally tend to preserve established practices, so incremental movement toward programmatic principles is both realistic and valuable [21]. 

#### Assuming Leadership Resistance

It is easy to assume that leaders are resistant to change, but this can be misleading. Leaders are not necessarily opposed to better approaches; rather, they may be cautious about disrupting systems that currently appear to function well. Strong student outcomes or high satisfaction levels can reinforce that caution. For this reason, advocates must work deliberately to build and sustain momentum for change. To successfully navigate leadership resistance, educators should employ established change management frameworks, such as Kotter’s eight steps [47]. Building a guiding coalition of both clinical faculty and senior administrative leaders is essential for the long term sustainability of a programmatic model.

A related pitfall is underestimating the resources required to provide effective student support. Programmatic assessment relies on proactive, personalised intervention, work that is labour intensive and cannot be delivered through traditional pastoral models or post failure tutoring alone. Support structures must therefore be redesigned, with specialist staff playing a central role.

In practice, limited faculty resources mean that adjusting every student’s progress individually using frequent feedback is a difficult goal to achieve. However, the message is not to increase the volume of work, but to shift the focus. By using technology and AI to automate data capture and highlight patterns, faculty can move away from repetitive marking toward high value, targeted interventions for those most in need. These capabilities are no longer optional; they are foundational [48].

#### Neglecting Cultural Shift: Faculty and Students

The final trap is failing to recognise the cultural shift required among faculty and students. Some educators assume that struggling students simply need to “work more”. Others believe that insufficient early support contributes to poor performance.

Programmatic assessment requires a different mindset. Learners develop at different rates across domains, and educators must understand individual trajectories. Personalised support becomes central. Students also need to move away from seeing assessment as a sequence of high stakes hurdles. They must view feedback as a core part of learning rather than a judgement of ability [16]. Without this shift, students may not engage with continuous assessment data or act on personalised guidance. Achieving these changes requires time and investment in data collection, analysis, and visualisation. In traditional systems, performance data often appears only at the end of the year and is not always shared. Programmatic assessment uses data continuously. It informs teaching, guides interventions, and supports timely feedback [4, 21]. The trap is using data only for end of year progression decisions rather than as a core tool for learning throughout the programme.

### Tips to Take on Board

Implementing programmatic assessment is complex. The following practical strategies can support a smoother transition:

1. Maintain programme level oversight. Keep assessment and curriculum decisions connected at the programme level. Avoid “module drift” where isolated changes undermine coherence. A full programme review, and ideally, programme-wide redesign, helps maintain constructive alignment, the principle that teaching activities and assessment methods are directly and transparently linked to intended learning outcomes, and ensures that learning outcomes map clearly onto assessment blueprints [24, 49].
2. Start small and expand gradually. Use pilots, then scale up. Incremental change reduces resistance and enables iterative refinement. Where possible, situate pilots within a programme level review rather than changing isolated assessments as this preserves constructive alignment, ensuring assessments remain matched to outcomes and learning objectives so the assessment blueprint remains coherent [50].
3. Prioritise faculty development. Build assessment literacy through workshops, calibration, and moderated decision making. Begin with foundational concepts such as reliability and validity, then progress to evidence supporting the case for change. To successfully navigate institutional change, consider employing established frameworks such as Kotter’s eight steps to build a guiding coalition of faculty and senior leaders. [33, 47] Approach staff with humility: provide training, resources, and practical exemplars that help them feel confident in the new approach.
4. Engage students early. Teach feedback literacy and help students understand the purpose of low stakes assessments and the aggregation of evidence. Recognise that students may be anxious about continuous visibility and showing less perfect work; explain the benefits of the cultural shift clearly to build trust [16].
5. Communicate the rationale clearly. Transparency builds trust. Share criteria, processes, and the way evidence is synthesised. Acknowledge how and why old systems are changing and reassure students and staff about the advantages. Use accessible dashboards and consistent messaging so students and staff understand what is changing, and why [50, 51].
6. Invest in student support structures and performance resilience. Deliver proactive, personalised support by ensuring that specialist staff, such as remediators, are well trained and equipped to respond to emerging student needs. Use the continuous flow of feedback to help students build performance resilience. This ensures that by the time they reach a high stakes national exam or patient crisis, their competence is well practised and stable, reducing the risk of choking under pressure [10, 52].
7. Use technology intelligently. Employ digital platforms for data capture and timely feedback. Utilise GenAI and Large Language Models (LLMs) to identify patterns and risk indicators while preserving human judgement. Ensure technology enables visibility across the programme, avoiding siloed module level perspectives [48, 52].
8. Protect time for feedback. High quality narrative feedback is central to programmatic assessment. Ensure it is timetabled, resourced, and supported. Reinforce that feedback is developmental rather than punitive, especially as students adjust to being assessed little and often [15, 33].
9. Monitor impact continuously. Collect stakeholder input, evaluate outcomes, and refine the approach over time. Programme level evaluation ensures that both intended and unintended consequences are visible, supporting an advice driven, inherently iterative and responsive implementation process [10, 50].

## Conclusion

Programmatic assessment reframes assessment as an ongoing, educationally purposeful process rather than an isolated judgement. It places feedback, reflection, and developmental growth at the centre, while maintaining credibility through robust programme level decision making. To build and sustain trust among learners and the public, systems must be transparent, coherent, and fair. Implementation inevitably requires cultural change for both faculty and students, organisational collaboration, and appropriately resourced support, and progress is likely to be incremental. Yet these challenges should not deter us. With clear leadership, intelligent use of technology (including GenAI and LLMs), and sustained attention to feedback quality and data literacy, programmatic assessment can strengthen learning, enhance equity, and produce graduates who are demonstrably competent and trusted by society.
